# Comparison of the asymmetries in muscle mass, biomechanical property and muscle activation asymmetry of quadriceps femoris between patients with unilateral and bilateral knee osteoarthritis

**DOI:** 10.3389/fphys.2023.1126116

**Published:** 2023-05-22

**Authors:** Weijian Chen, Congcong Li, Yi Wang, Xingxing Shen, Zugui Wu, Junyi Li, Zixuan Ye, Ruian Xiang, Xuemeng Xu

**Affiliations:** ^1^ The Fifth Clinical College of Guangzhou University of Chinese Medicine, Guangzhou, China; ^2^ Guangdong Provincial Second Hospital of Traditional Chinese Medicine, Guangzhou, China

**Keywords:** knee osteoarthritis, asymmetry, muscle thickness, shear modulus, surface electromyography

## Abstract

**Background:** More and more attention has been paid to the research of muscle mass and muscle quality of quadriceps femoris (QF) in knee osteoarthritis (KOA). This study aimed to explore the asymmetric changes of muscle mass, biomechanical property and muscle activation in the inter-limbs QF of KOA patients, and tried to provide a novel insight for the evaluation, prevention and treatment of KOA.

**Methods:** A total of 56 Participants with unilateral or bilateral KOA were included in this study: 30 patients with unilateral pain and 26 patients with bilateral pain were assigned to the bilateral group (BG) and unilateral group (UG), respectively. The symptom severity of bilateral lower limbs was evaluated by visual analogue scale, and the relatively serious leg (RSL) and relatively moderate leg (RML) were classified. The thickness of rectus femoris (RF), vastus intermedius (VI), vastus medialis (VM) and vastus lateralis (VL) were measured by ultrasound. The Shear wave elastography (SWE) techniqie was used to measure the shear modulus of RF, VM and VL. Surface electromyography (sEMG) was used to assess the root mean square (RMS) of the RF, VM, and VL during straight leg raising in a sitting position and squatting task. We calculated the asymmetry indexes of inter-limbs for the corresponding indices of the measured muscles.

**Result:** Thickness of RF, VI and VL of RSL was lower than those on RML (*p* < 0.05), and thickness of VM was lower more significant (*p* < 0.01). Thickness of RF, VI and VL of RSL was also lower than those of RML in BG (*p* < 0.05), however, there was no significant difference in VM thickness (*p* > 0.05). There were no significant difference in Asymmetry indexes of all measured muscle thickness between the two groups (*p* > 0.05). The Shear modulus of RF, VM, and VL in the RML of UG and BG was higher than those in the RSL (*p* < 0.05). In sitting and straight leg raising task, the RMS of RF, VM and VL in RML were higher than those in RSL, UG and BG both showed this trend (*p* < 0.05). About squatting task, in UG, the RMS of the three muscles in RML of patients were also higher than those in the RSL (*p* < 0.05). However, the difference was not significant in BG (*p* > 0.05). In the straight leg raising task, the asymmetry indexes of RMS in RF, VM, and VL of both the two groups were positively correlated with VAS scores (*p* < 0.05).

**Conclusion:** The muscle thickness, shear modulus and muscle activation electromyography of QF in RML were higher than those of RSL in unilateral KOA patients. The VM of RML in bilateral KOA patients may show muscle thickness degeneration earlier, which is closer to the VM of RSL. The shear modulus of RF, VM, and VL were higher on the RML side during the single-leg task, but there may be passive compensation for muscle activation in both lower limbs during the bipedal task. In conclusion, there is a general asymmetry of QF muscle mass, biomechanics Characteristic and performance in patients with KOA, which may provide new ideas for the assessment, treatment and rehabilitation of the disease.

## Introduction

Knee osteoarthritis (KOA) is a very common degenerative joint disease in the elderly (Sharma, 2021), the most common cause of mobility limitation in adults (Katz et al., 2021). Pain and functional limitations caused by KOA negatively affect the quality of daily life (Felson et al., 2000). With the aging of the population, the prevalence of KOA is getting higher and higher, which brings a huge economic burden to both individuals and society (Cowan et al., 2015; Cui et al., 2020). Osteoarthritis (OA) has long been visualized as a “wear and tear” disease, but modern medicine is more aware of the complex pathophysiology that affects multiple joints and periarticular structures (Katz et al., 2021). KOA is primarily driven by a combination of biomechanical and inflammatory alterations that cause changes in the joints and surrounding muscles (Griffin and Guilak, 2005; Briggs-Price et al., 2022). As the “power and control device” of joint activity, muscle is a link that can not be ignored in the study of the occurrence and development of KOA (Culvenor et al., 2017; Zeng et al., 2022). Weakened muscle strength is closely related to structural degeneration and symptom development in KOA, even the muscle lesions appear earlier than the joints (Thomas et al., 2010).

As the largest and strongest muscle group of the lower extremities, the quadriceps femoris (QF) is an important part of the knee extensor mechanism. The QF consists of four muscles:rectus femoris (RF), vastus intermedius (VI), vastus medialis (VM) and vastus lateralis (VL). As the stabilizer and shock absorber of the knee, QF can dissipate the harmful load on the knee to a certain extent and help to maintain the mechanical environment of the joint (Øiestad et al., 2015). Previous studies have shown that QF strength is decreased and muscle cross-sectional area is reduced in KOA patients (Aslan et al., 2020; Yamauchi et al., 2020). In patients with knee osteoarthritis, changes in muscle strength and shape may be affected by many factors. Such as pain (Ruhdorfer et al., 2014), changes in muscle structure (Mohajer B), decreased physical activity, changes in joint load, obesity, poor alignment, trauma and joint instability. Therefore, it seems necessary to determine the current fixed muscle mass to assess muscle quality. Ultrasound (US) can clearly distinguish muscles, fat, bone and other structures. It has been reported that it can be applied to the evaluation of quadriceps femoris muscle, which is cheap, practical and safe (Noorkoiv et al., 2010). Measurement the muscle layer thickness by US is considered one of the methods to assess the muscle mass of QF (Koca et al., 2014).

Shear wave elastography (SWE) is a novel ultrasonography techniqie. It transmits and tracks shear waves to obtain Shear modulus values, and dynamically depicts the distribution of SWE in target tissues in real time (Niu et al., 2022). Shear modulus value can be used to quantify local muscle stiffness (Chalchat et al., 2022), and tiffness is a proxy for the force of muscle contraction (Lin et al., 2022). Evaluating the passive and active mechanical properties of QF can reflect the contractile performance of its muscles. Surface electromyography (sEMG) is widely used to assess muscle activation during isometric and dynamic actions of the limbs and trunk (Naik et al., 2017). RMS is an important index of sEMG, which refers to the integrated electromyography value divided by the time of measuring the integrated electromyography. It can reflect the effective value of nerve muscle fiber discharge, which is related to motor unit recruitment and excitatory rhythm synchronization (Pullman et al., 2000).

sEMG has been widely used to evaluate lower limb muscle function and coordination in KOA patients, and ultrasound has also been used to observe muscle structure to evaluate muscle mass and morphology. The use of Shear wave elastography to evaluate muscle biomechanical properties is still rare. This study was the first to comprehensively use the above equipments to evaluate the muscle quality, biomechanical properties and muscle contraction force of QF in patients with KOA, and to explore the correlation between the observed indicators. Based on the above equipment and indicators, we designed this study to comprehensively evaluate the muscle mass, biomechanical properties and muscle contractility of QF in KOA patients. In addition, we try to explore the differences of asymmetry between unilateral and bilateral patients in these characteristics, so as to provide some ideas for the prevention and treatment of KOA.

## Methods

### Participants

All the participants of this study were rucruited from the Department of Orthopedics, Guangdong Second Traditional Chinese Medicine Hospital. The inclusion criteria were as the following: 1) the age range were 45–70 years old, 2) BMI ≤ 30 kg/m^2^, 3) KOA diagnosed by the American College of Rheumatology clinical criteria, 4) Kellgren/Lawrence (K/L) grade ≥ 2 in one or two knees, 5) an ability to complete the half-squat movement for 20 s in a single session. Exclusion criteria for KOA patients included the following: 1) concomitant neurological disorders such as stroke, spinal related disorders, vertigo or Parkinson’s disease, 2) history of major trauma, surgery or deformity of the lower limbs, 3) any medication that affects the tension, stiffness and other properties of muscles and tendons. 4) Strenuous exercise had been performed 48 h before the test, and 5) Other inflammatory arthritis.

A total of 56 subjects with unilateral or bilateral KOA were included: 30 patients with unilateral pain made up the unilateral group (UG), and 26 patients with bilateral pain made up the bilateral group (BG). The severity of pain symptoms was assessed according to the visual analogue scale (VAS), and the symptomatic leg (in UG) or the more symptomatic leg (in BG) was defined as the relatively serious leg (RSL) and the contralateral leg as the relatively moderate leg (RML). The research team had collected all participants’ demographic and clinical information, including their name, gender, age, height, weight and duration of diseasedisease, etc.

### Measurement of the thickness of the quadriceps

The measurement was performed by experienced sonographers using Supersonic Imaging Aixplorer (French) color Doppler ultrasound diagnostic instrument (Supplementary Figure S1). The thickness of RF, VI, VM, VL, and thigh subcutaneous fat (SF) were measured. Measurement method: L15-4 linear array probe was selected, the frequency was 4–12 MHz (Aily et al., 2019; Chopp-Hurley et al., 2020; El-Ansary et al., 2021), and the ultrasonic mode was set as muscle inspection mode. The subject was placed in supine position, legs relaxed and straight, without internal and external rotation of the hip and knee. The probe was vertically placed at the middle and lower 1/3 of the upper margin of the anterior superior iliac spine and patella, and the probe was pointed to the dorsal side, so that the sound beam was displayed and perpendicular to the femur body. Then the position of the probe was adjusted horizontally so that the image showed RF and VI at the same time. The thickness of RF and VI was measured at 10 cm above the patellar area. The VL thickness was measured at 10 cm supratellar and 18° long axis of femur. The VM thickness was measured at 10 cm supratellar and 55° inside the long axis of the femur. After freezing the images, we measured the thickness of the corresponding muscle with the built-in measurement system of the US. Each data was measured 3 times and its average value was taken. Due to the shallow position of the QF, the ultrasonic probe should be placed gently and vertically on the skin surface during operation to avoid the compression of soft tissue and the measurement error caused by the Angle between the probe and the skin contact surface (Zheng et al., 2019).

### Measurement of shear modulus

Keep the above measuring position and switch to the color Doppler ultrasonography to SWE measuring mode. Eby SF et al. selected the upper limb muscles of pigs as study samples and found that the accuracy of SWE measurements was higher when the probe was parallel to the long axis of the sample muscle (Eby et al., 2013). Based on the muscle thickness measurement points, the probe was rotated to the parallel direction of the muscle for observation. The muscle was divided into three parts: upper, middle and lower layers. A fixed-size square region (SWE Box) was placed in the middle layer, to delimit the elastographic field of view. The SWE box can visualize and analyze the propagation of shear-wave in the muscle. Waited about 5 s for the SWE Box to be filled with stable and uniform shear-wave. Then freezed the image, enabled the dedicated analysis plugin (Q-Box, Supersonic Imaging), set the measurement area to a circle with a diameter of about 5 mm, and try to select a more uniform area covered by SWE. The analysis plugin automatically calculated the Shear modulus values (mean) of muscle tissue in the Q-BOX. Samples with large numerical bias due to poor filling effect, excessive probe pressure, excessive muscle contraction and other reasons were excluded. For each freeze-frame image, the mean value of two qualified observation points was taken for statistical analysis. RF, VM, and VL were measured sequentially.

### Measurement of muscle sEMG

The surface electromyography telemetry system (Italy, model: BTS S. P. A, FreeEMG1000, software version:FreeEMG-3.3.7.0) was used for testing. Six channels were used to record the sEMG signals of the RF, VM and VL of both lower limbs, respectively. Parameter Settings: preamplifier, input impedance>100 MOhm, common mode rejection ratio greater than 110 dB, channel sampling bandwidth from 20 to 400 Hz, and sensitivity of 1 mV. The frequency of EMG data acquisition was 1000 Hz. Before attaching the electrode, the hair was scraped, the skin was wiped with fine sandpaper and 75% medical alcohol, and the sweat at and around the electrode site was wiped with a gauze block. Electrode placement: ① RF: electrodes were placed at the midpoint of the line between the superior edge of the patella and the anterior superior iliac spine in front of the thigh; ② VM: the electrode was placed at 20% of the distance between the medial space of the knee joint and the anterior superior iliac spine, and the Angle between the connecting line of the two electrodes and the long axis of the femur was 55°; ③ VL: the electrodes were placed at 1/10 of the distance from the lateral space of the knee joint to the anterior superior iliac spine above the superior lateral corner of the patella, and the Angle between the connecting line of the two electrodes and the long axis of the femur was 15°. The center distance between the two electrodes was 2 cm. Test actions: ① straight leg raising task in sitting position: the subject sat on a chair about 40 cm high, and the lower limb muscles relaxed. After recording sEMG signal, the subject raised the test lower limb slightly off the chair for 15 s, and the order was left lower limb first and then right lower limb. ② Squatting task: the subjects’ feet were at shoulder level and the tips of their feet were forward. After the sEMG signal began to be recorded, the subjects began to squat and stopped when the squat reached the limit. Measurements were repeated three times for each movement, with a 5-min rest between each measurement. Processing of surface electromyography data: The raw electromyography data were rectified, filtered and smoothed by analysis software, and the root mean square (RMS) value was calculated (Supplementary Figure S2). RMS were averaged over three cycles of repeated measurements.

### Calculation of the asymmetry indexes

The asymmetry index (Supplementary Method S1) of muscle thickness and Shear modulus were calculated according to previous studies (Chen et al., 2021). The thickness asymmetry indexes of RF, VI, VM, and VL were denoted by Asythick (RF), Asy-thick (VI), Asythick (VM), Asythick (VL), respectively. The Shear modulus asymmetry indexes of RF, VM, and VL were expressed using Asy-μ (RF), Asy-μ (VM), and Asy-μ (VL). Asy-RMS (RF), Asy-RMS (VM), and Asy-RMS (VL) were used to reprepresent the RMS asymmetry index of RF, VM, and VL.

### Statistical analysis

Statistical analyses were performed using SPSS 22.0 software (IBM, Corp., NY, United States). Continuous characteristics of the study were checked for normality using the Shapiro–Wilk test. Homogeneity of variances was tested by Levene’s test. Measurement that conforms to the normal distribution is represented as *x* ± s deviation, and non-normally distributed measurement data are represented as medians and interquartile ranges. According to the results of the normality test, the paired Stusent *t*-test was used for normal distribution in the same group, and independent *t*-test was used to compare the differences between the two groups. Non-normally distributed measurement data were compared by the non-parametric test (Mann–Whitney). Numerical data are represented by rates; and compared by the χ^2^ test. Spearman correlation coefficients were used to analyze the correlations between muscle thickness asymmetry index, muscle RMS asymmetry index and VAS score. Statistical significance level was accepted at *p* < 0.05.

## Result

### Participants characteristics

In a preliminary study, 14 subjects were selected and the effect size of asymmetry index of RF muscle thickness between bilateral KOA patients and unilateral KOA patients was 0.77, Taking α at 5% and power at 80%, the estimated sample size was 22 subjects per group. Following the inclusion and exclusion criteria, 56 patients were included in the final analysis. The information collected indicates no statistically significant differences in sex, age, height, weight, BMI, duration of disease, VAS score among both groups. The basic characteristics of all participants is shown in Table 1.

**TABLE 1:** Demographic characteristics of the study patients.

	UG (*n* = 30)	BG (*n* = 26)	*p*-value
Male/Female	7/23	4/22	0.455
Age (year)	68.13 ± 9.44	65.08 ± 8.90	0.220
Heigh (cm)	159.80 ± 6.61	157.80 ± 6.92	0.259
Weigh (kg)	61.97 ± 6.65	62.15 ± 7.93	0.924
BMI (kg/m^2^)	24.32 ± 2.70	25.00 ± 3.10	0.383
Duration (year)	3.25 ± 3.00	5.16 ± 4.19	0.053
VAS (RSL)	5.70 ± 1.44	6.31 ± 1.12	0.088

UG, unilateral group; BG, bilateral group; BMI, body mass index; RSL, relatively severe leg.

### Muscle thickness analysis

As was shown in Table 2, the results revealed that in the UG, thickness of RF, VI, and VL of RSL was lower than those on RML (*p* < 0.05), and thickness of VM was lower more significant (*p* < 0.01). Thickness of RF, VI, and VL of RSL was also lower than those of RML in BG (*p* < 0.05), however, there was no significant difference in VM thickness (*p* > 0.05). There were no significant differences in Asythick (RF), Asythick (VI), Asythick (VM), and Asythick (VL) between the two groups (*p* > 0.05), as shown in Table 4.

**TABLE 2:** Thickness of the quadriceps femoris muscle (cm).

Group	UG		*p*-value	BG		*p*
Side	RSL	RML		Value	RML	
RF	0.99 ± 0.16	1.05 ± 0.21	0.023^*^	0.92 ± 0.14	0.97 ± 0.17	0.013^*^
VI	1.03 ± 0.20	1.11 ± 0.26	0.013^*^	0.92 ± 0.14	0.97 ± 0.14	0.030^*^
VM	3.52 ± 0.18	3.73 ± 0.21	0.000^**^	3.49 ± 0.23	3.55 ± 0.16	0.078
VL	1.42 ± 0.14	1.48 ± 0.14	0.031^*^	1.40 ± 0.16	1.44 ± 0.16	0.039^*^

UG, unilateral group; BG, bilateral group; RSL, relatively severe leg; RML, relatively moderate leg; RF, rectus femoris; VI, vastus intermedius; VM, vastus medialis; VL, vastus lateralis; * indicates *p* < 0.05; ** indicates *p* < 0.01.

### SWE analysis

The Shear modulus of RF, VM, and VL in the RML of UG patients was significantly higher than those in the RSL (*p* < 0.01). In BG, the Shear modulus of RF, VM, and VL of RML was also higher than those of RSL (*p* < 0.05). All data are presented in Table 3 as follows. In the comparison of the asymmetric index of the shear modulus of the lower limbs between the two groups, there were all significant differences in RF, VM, and VL. And the shear modulus asymmetry indexes of the three muscles in UG were significantly higher (*p* < 0.01), as shown in Table 4.

**TABLE 3:** Comparison of muscle Shear modulus between two groups.

Muscle	UG		*p*	BG		*p*
Side	RSL	Value		value	RML	
RF	8.19 ± 1.65	9.94 ± 1.55	0.000^**^	7.35 ± 1.30	7.94 ± 1.44	0.041^*^
VM	7.63 ± 1.26	9.19 ± 1.72	0.000^**^	7.06 ± 0.95	7.48 ± 1.08	0.024^*^
VL	7.69 ± 1.07	9.33 ± 1.22	0.000^**^	7.26 ± 1.47	7.75 ± 0.99	0.029^*^

UG, unilateral group; BG, bilateral group; RSL, relatively severe leg; RML, relatively moderate leg; RF, rectus femoris; VM, vastus medialis; VL, vastus lateralis; * indicates *p* < 0.05; ** indicates *p* < 0.01.

**TABLE 4:** Comparison of asymmetry indexes about muscle thickness and shear modulus between the two groups.

	UG	BG	*p*-value
Asythick (RF)	9.12 (4.24, 15.31)	5.27 (2.32, 9.81)	0.053
Asythick (VI)	10.17 (4.42, 17.97)	6.88 (4.46, 13.58)	0.175
Asythick (VM)	6.16 (3.57, 9.79)	4.49 (3.49, 6.21)	0.073
Asythick (VL)	6.04 (3.03, 11.19)	4.37 (2.26, 7.00)	0.097
Asy-μ (RF)	18.46 (12.41, 31.25)	10.14 (5.65, 20.22)	0.025^*^
Asy-μ (VM)	15.56 (11.39, 22.79)	8.39 (5.13, 14.64)	0.004^**^
Asy-μ (VL)	18.84 (14.33, 28.02)	8.99 (2.99, 14.25)	0.000^**^

UG, unilateral group; BG, bilateral group; Asythick (RF), asymmetry index of rectus femoris thickness; Asythick (VI), asymmetry index of vastus medialis thickness; Asythick (VM), asymmetry index of vastus medialis thickness; Asythick (VL), asymmetry index of vastus lateralis thickness; Asy-μ (RF), asymmetry index of rectus femoris’s Shear modulus; Asy-μ(VM), asymmetry index of vastus medialis’s Shear modulus; Asy-μ (VL), asymmetry index of vastus lateralis’s Shear modulus; * indicates *p* < 0.05; ** indicates *p* < 0.01.

### sEMG analysis

**FIGURE 1:**
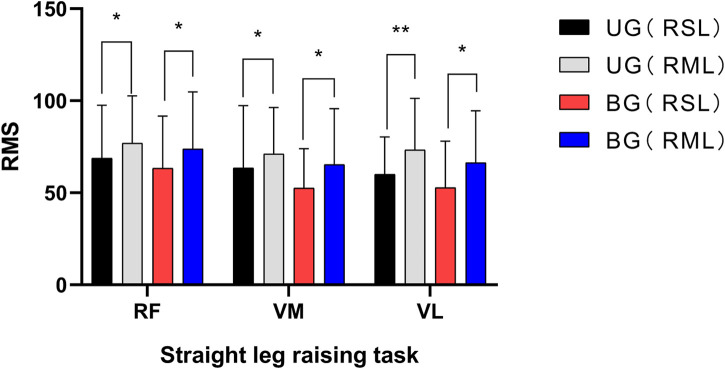
UG, unilateral group; BG, bilateral group; RSL, relatively severe leg; RML, relatively moderate leg; RF, rectus femoris; VM, vastus medialis; VL, vastus lateralis; * indicates *p* < 0.05; ** indicates *p* < 0.01.

**FIGURE 2:**
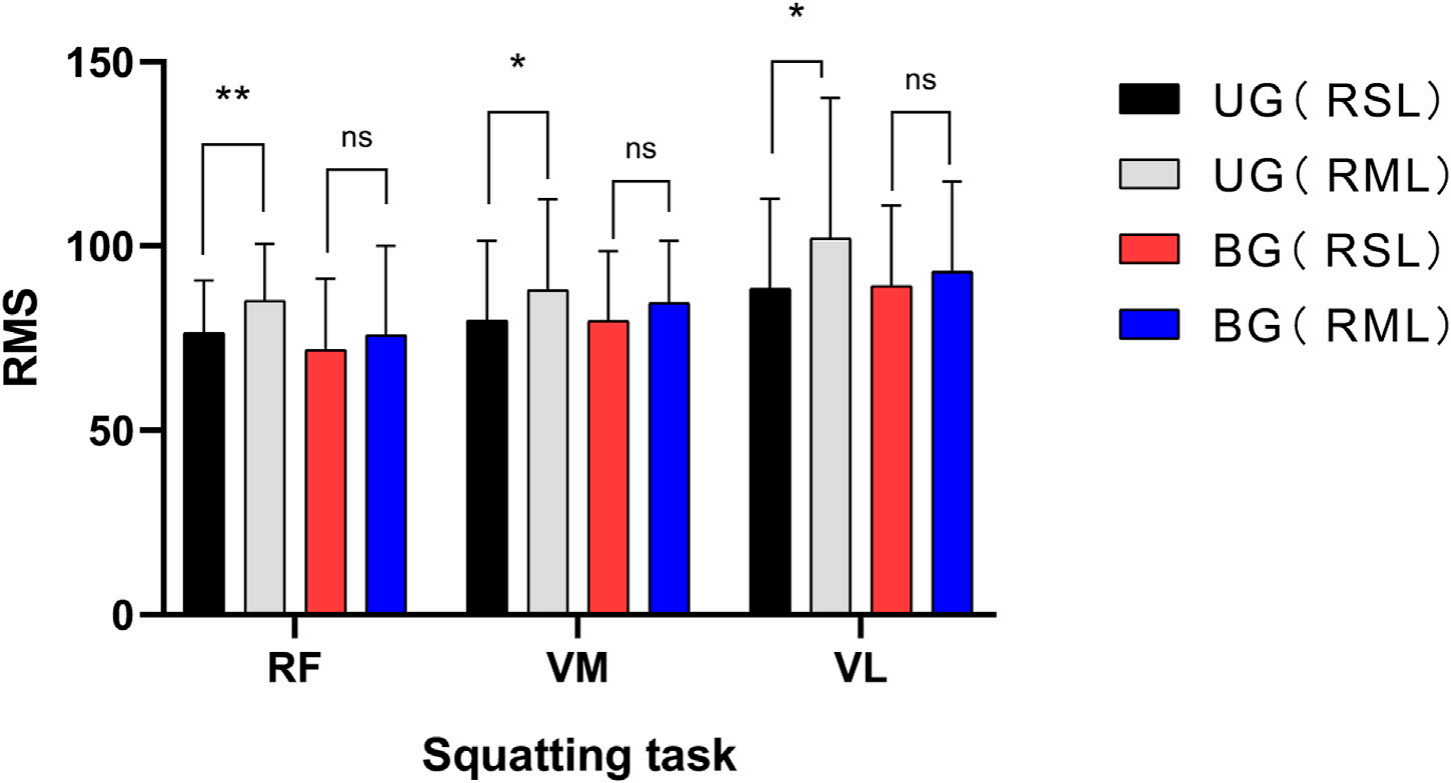
UG, unilateral group; BG, bilateral group; RSL, relatively severe leg; RML, relatively moderate leg; RF, rectus femoris; VM, vastus medialis; VL, vastus lateralis; * indicates *p* < 0.05; ** indicates *p* < 0.01; ns, no significance.

In sitting and straight leg raising task, the RMS of RF, VM, and VL in RML were higher than those in RSL, UG and BG both showed this trend, (*p* < 0.05), as shown in Figure 1. About squatting task, in UG, the RMS of the three muscles in RML of patients were also higher than those in the RSL (*p* < 0.05). However, the difference was not significant in BG (*p* > 0.05), shown in Figure 2. The asymmetry index of RMS was further compared between the two groups of muscles in different tasks. In the squatting task, the RMS asymmetry index of the RF, VM, and VL in the UG showed a trend toward higher. Among them, the differences in VM and VL were significant (*p* < 0.05, *p* < 0.01, respectively). However, the differences between the two groups were not significant in the task of straight leg raising in sitting position. Table 5.

**TABLE 5:** Comparison of asymmetry indexes about muscle sEMG (RMS) between the two groups.

Task	Muscle	UG	BG	*p*-value
straight	Asy_RMS_ (RF)	22.61 (16.66, 25.60)	20.57 (10.85, 38.79)	0.693
leg raising	Asy_RMS_ (VM)	18.73 (10.91, 26.66)	20.88 (14.63, 39.33)	0.286
	Asy_RMS_ (VL)	19.14 (7.15, 38.99)	29.07 (15.43, 37.51)	0.287
squatting	Asy_RMS_ (RF)	19.36 (12.16, 25.92)	11.95 (6.41, 19.60)	0.057
	Asy_RMS_ (VM)	19.60 (9.64, 26.17)	8.23 (4.61, 15.55)	0.015^*^
	Asy_RMS_ (VL)	32.13 (11.97, 50.81)	9.90 (7.16, 21.05)	0.002^**^

UG, unilateral group; BG, bilateral group; Asythick (RF), asymmetry index of rectus femoris thickness; Asythick (VI):asymmetry index of vastus medialis thickness; Asythick (VM), asymmetry index of vastus medialis thickness; Asythick (VL), asymmetry index of vastus lateralis thickness Asy-_RMS_(RF), asymmetry index of rectus femoris’s RMS; Asy-_RMS_(VM), asymmetry index of vastus medialis’sRMSs; Asy-_RMS_(VL), asymmetry index of vastus lateralis’sRMS; * indicates *p* < 0.05; ** indicates *p* < 0.01.

### Correlation analysis between muscle thickness asymmetry index, muscle sEMG asymmetry index, and VAS score

There war no significant correlation between the thickness asymmetry index of RF, VI, VM, and VL in BG and VAS score. However, in UG, the trend of positive correlation between muscle thickness and VAS score was more obvious. The correlation between Asythick (VM), Asythick (VI) and VAS score was statistically significant (*p* < 0.05), and Asythick (RF), Asythick (VL) were not statistically significant (*p* = 0.090, *p* = 0.084, respectively). In the straight leg raising task, the asymmetry index of RMS in RF, VM and VL of both the two groups were positively correlated with VAS scores (*p* < 0.05). (All data are presented in Supplementary Table S1 as follows)

## Discussion

The occurrence and development of KOA have a serious impact on the muscle groups around the knee joint. After the muscle function is affected, the joint degeneration and muscle weakness of KOA patients will be further aggravated, and even muscle atrophy will occur, which is a vicious circle (Bennell et al., 2011; Bennell et al., 2013). And Thomas et al. found that the decrease of QF muscle abdominal cross-sectional area existed before the occurrence of KOA, which further confirmed the correlation between the decrease of muscle cross-sectional area and the incidence of KOA (Thomas et al., 2010). The main molecular mechanisms of muscle atrophy include decreased protein synthesis, increased breakdown, and impaired muscle satellite cell regeneration (Cohen et al., 2015). However, a study by Noehren et al. showed that the hypofunction of QF in KOA patients may not be caused by muscle fiber size damage, but by extracellular matrix expansion (Noehren et al., 2018). In addition, impaired satellite cell density, high profibrotic gene expression and Ⅰ-to-Ⅱ (slow-to-fast) fiber type switching may also contribute to the decreased muscle quality in KOA patients (Noehren et al., 2018). A study has found that compared with healthy people, only VM in QF showed significant decrease in muscle thickness in mild KOA patients, and other differences were not obvious (Taniguchi et al., 2015). However, the content of different types of muscle fibers may change, which has a negative impact on muscle quality and the cooperative contraction of quadriceps femoris is abnormal. Therefore, in the early and middle stages of KOA, it may not be comprehensive and accurate to judge muscle quality and function simply by muscle thickness or cross-sectional area.

In this study, the thickness of RF、VI、VM, and VL of RSL in UG was lower than those of RML. In BG, only the difference in the thickness of the VM between the two lower limbs was not significant, but the thickness of RF, VM, and VL was higher in RML. There was a significant reduction of QF thickness in RSL relative to RML, which may be related to more severe long-term pain, functional limitation, and load reduction (Tsukada et al., 2020). Several previous studies in human have suggested unilateral mechanical loading as a possible cause of muscle atrophy. In the model of single lower limb loading, the contralateral lower limb muscles would show disuse atrophy, and the longer the time was, the more serious the muscle reduction would be (Schulze et al., 2002; Al-Khlaifat et al., 2016). VM is the latest muscle to develop and the weakest muscle in quadriceps phylogenies. Therefore, disuse atrophy occurs first in VM after being affected by factors such as injury, immobilization, or surgery (Fox, 1975). Although the RML symptoms of BG patients are relatively mild, VM of BG patients may have different degrees of muscle atrophy, similar to RSL, because it is also the affected limb. The muscle thickness of RF, VI, and VL in RML may be significantly better than that in RSL because of the shorter course of disease and lower K/L grade. This is similar to the view of Taniguchi et al. (Taniguchi et al., 2015). In this study, there was no significant difference in asymmetry index of bilateral quadriceps thickness between BG and UG groups. This suggests that the quadriceps thickness asymmetry in BG is also evident. A study by Lee et al. used dual-energy X-ray absorptiometry to measure lower limb muscle mass in KOA patients. The asymmetry index of both lower limbs was then calculated (Lee et al., 2019). It was found that the asymmetry index was correlated with knee pain and significantly higher grade of radiographic KOA in male patients. However, there was no significant correlation in female patients. However, even in healthy elderly women, the asymmetry of lower limb muscles is more obvious than that of men (Lee et al., 2019; Mertz et al., 2019). It can be seen that the causes of this muscle mass asymmetry are complex and not only caused by unilateral or bilateral KOA. Therefore, training interventions should be used to enhance muscle strength and power to maintain and improve lower extremity function, while reducing asymmetric parameters is not so important (Mertz et al., 2019).

In order to conduct more accurate SWE assessment, RF, VM, and VL of the superficial layer of the QF were selected for measurement in this study. The SWE of the three muscles in the RML was higher than that in the RSL in both groups. In addition, the shear modulus asymmetry index of UG’s RF、VM, and VL were significantly higher than BG’s. Thus, it indicated that the leg with KOA was decreased by different degrees of muscle SWE compared with the healthy leg. It can be seen that the SWE of the muscles in the leg with KOA is lower than that in the healthy leg. The vicious cycle of “muscle spasm-pain- muscle spasm” is common in muscle diseases, which easily leads to increased muscle tone and stiffness (Bentley, 1996; van Dieën et al., 2003; Bialas et al., 2019; Maughan and Shirreffs, 2019). But the quadriceps muscle mass reduction and muscle weakness are the most common peripheral muscle disorders in patients with KOA (Slemenda et al., 1997). The pain mainly concentrated in the knee joint and around the tibial plateau, and the quadriceps femoris had relatively less pain in the early stage of KOA. Some studies have assessed the risk of muscle weakness and sarcopenia in COPD patients by measuring the RF SWE (Deng et al., 2021; Niu et al., 2022). The results showed that the shear modulus of patients was significantly lower than that of normal people, and the sensitivity of this index was higher than that of muscle thickness and cross-sectional area. The decrease in muscle stiffness could reflect muscle fiber atrophy (Wen et al., 2018) or a combination of muscle edema, inflammation or lipid accumulation, and fiber atrophy (Alfuraih et al., 2019). What’s more, SWE was independent of sex, height, and body mass, but muscle cross-sectional area and thickness are affected by height and weight (Deng et al., 2021). Therefore, SWE plays a role in evaluating the biomechanical property of QF and measuring muscle quality in KOA patients. Improving the SWE of quadriceps femoris muscle by intervention may have a positive effect on the prevention and treatment of KOA.

As expected, the RMS of the measured muscles in the RML was significantly lower in both groups during the sitting and leg raising task. In the squatting task, the RMS of muscles in the UG RSL was also significantly lower. While the three muscles in BG showed the same trend, but the differences were not significant. In bipedal tasks, the placement of body weight mostly through the unaffected or minor affected limb has been thought as one of the compensation strategy expected for KOA patients (Petrella et al., 2017). However, some studies evaluating balance function found that patients with bilateral KOA showed significant postural sway (Khalaj et al., 2014). Moreover, the adjustment strategy in the squatting task is complex (Hase et al., 2004), increasing the fear of pain (Vincent and Vincent, 2012) and the attempt to maintain postural control (Petrella et al., 2017). Thus, there may also be more passive activation of the lower limb muscles of the RSL in this unstable state. Eliminating inter-limbs sEMG asymmetry on the basis of improving the overall recruitment of motor units of both lower limbs may be beneficial for KOA. It can be noted that there is no significant difference in bilateral VI and VL in BP patients, but RMS of all three muscles in RML were significantly higher in the single-leg open kinetic chain task. It indicates that muscle thickness may not absolutely reflect muscle quality and function in already injured muscles. Combined with electrophysiological analysis of muscle activity, muscle contraction and activation ability can be analyzed in addition to muscle mass.

As mentioned above, the straight leg raising task in the sitting position may eliminate the interference of body instability and provide a relatively more accurate assessment of the actual muscle activation ability of the unilateral limb. The muscle sEMG asymmetry index in this task were significantly positively correlated with the degree of pain, and this was true in both groups. The UG muscle thickness asymmetry index is positively correlated with the degree of pain, which also supports that the more severe the symptoms of unilateral KOA patients, the affected limb may suffer from more serious muscle degeneration. In BG, only VM thickness asymmetry index is significantly positively correlated with the degree of pain, highlighting the initial and importance of VM degeneration in KOA, which needs to be paid attention to. The above asymmetry index may provide certain reference significance for the evaluation of the severity and treatment of KOA.

### Limation

There are some limitations in our study that need to be considered. Firstly, because of lack of a healthy control group, we were not sure whether the muscle mass, biomechanical properties and muscle contractility were changed during KOA degeneration. Muscle assessment should be performed in healthy people to clarify whether factors such as amount of lower limb activity and differences in dominant foot have an effect on these muscle characteristics and lower limb asymmetry. Secondly, because of the wide age range of the included participants, the degree of physiological muscle degeneration may interfere with the comparison of interindividual differences. This is one of the reasons why we further carried out the lower limb asymmetry index comparison. What’s more, some KOA patients have great differences in the K/L grade and course of disease between the two sides. However, we only included patients with KOA who could cooperate with the whole sitting straight leg raising and squatting task. This indicates that there were no samples with poor lower limb function that would greatly interfere with the analysis. In addition, in this study, VAS score was used to distinguish RSL and RML, which considered that severe symptoms would affect the use of lower limbs and have graeter impact on muscles, but might ignore the mechanical effect of knee joint structural degeneration. Despite the difficulties and shortcomings, this study has obtained preliminary results, and more participants will be enrolled and further in-depth analysis will be conducted in the future.

## Conclusion

The muscle thickness, shear modulus and muscle activation electromyography of QF in RML were higher than those of RSL in unilateral KOA patients. The VM of RML in bilateral KOA patients may show muscle thickness degeneration earlier, which is closer to the VM of RSL. The shear modulus of RF, VM, and VL were higher on the RML side during the single-leg task, but there may be passive compensation for muscle activation in both lower limbs during the bipedal task. Our study found that the muscle thickness, shear modulus and muscle activation electromyography of QF in RML were higher than those of RSL in unilateral KOA patients. The VM of RML in bilateral KOA patients may show muscle thickness degeneration earlier, which is closer to the VM of RSL. The shear modulus of RF, VM, and VL were higher on the RML side during the single-leg task, but there may be passive compensation for muscle activation in both lower limbs during the bipedal task. This asymmetry in KOA patients found in our study may provide ideas for the assessment, treatment and rehabilitation of the disease, such as used for early evaluation of muscle parameters and formulation of corresponding muscle intervention plan guiding functional exercise.

## Data Availability

The original contributions presented in the study are included in the article/Supplementary Material, further inquiries can be directed to the corresponding author.
