# Supplementary material for: Comparison of the asymmetries in muscle mass, biomechanical property and muscle activation asymmetry of quadriceps femoris between patients with unilateral and bilateral knee osteoarthritis
Source: Front Physiol. 2023 May 22;14:1126116. doi: 10.3389/fphys.2023.1126116 (PMC10239935; doi:10.3389/fphys.2023.1126116)
Supplement: Supplementary file 1 [file DataSheet1.docx]

**Supplemental Appendix**

**Method 1**

$Asy(\%)=\left( \frac{V_{larger}-V_{lower}}{V_{larger}} \right)\times100$

asymmetry index was calculated using this equation.

*Asy:* asymmetery index; *V_larger_*: larger value; *V_lower_*: lower value.

**Figure1**

**
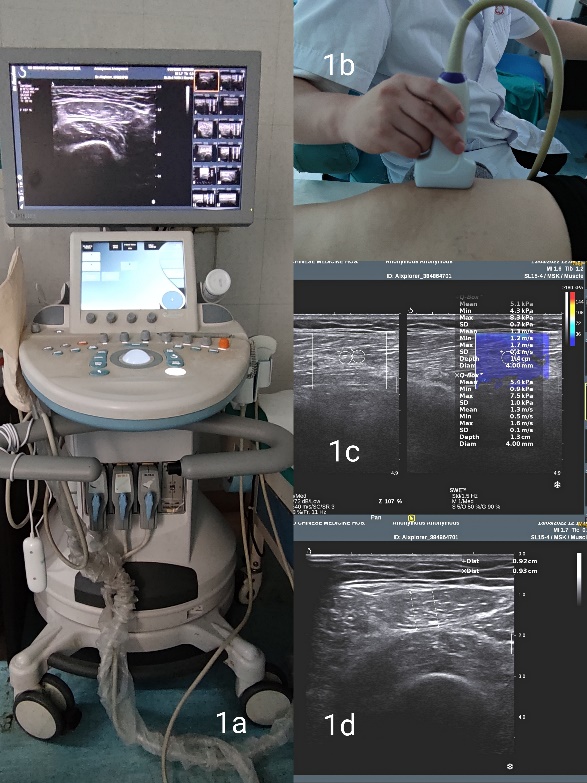
**

1a: Supersonic Imaging Aixplorer(French) color Doppler ultrasound diagnostic instrument

1b: Expert sonographers use ultrasound to perform muscle measurements

1c: Measurements in SWE mode

1d: Measurement of muscle thickness

**Figure 2**


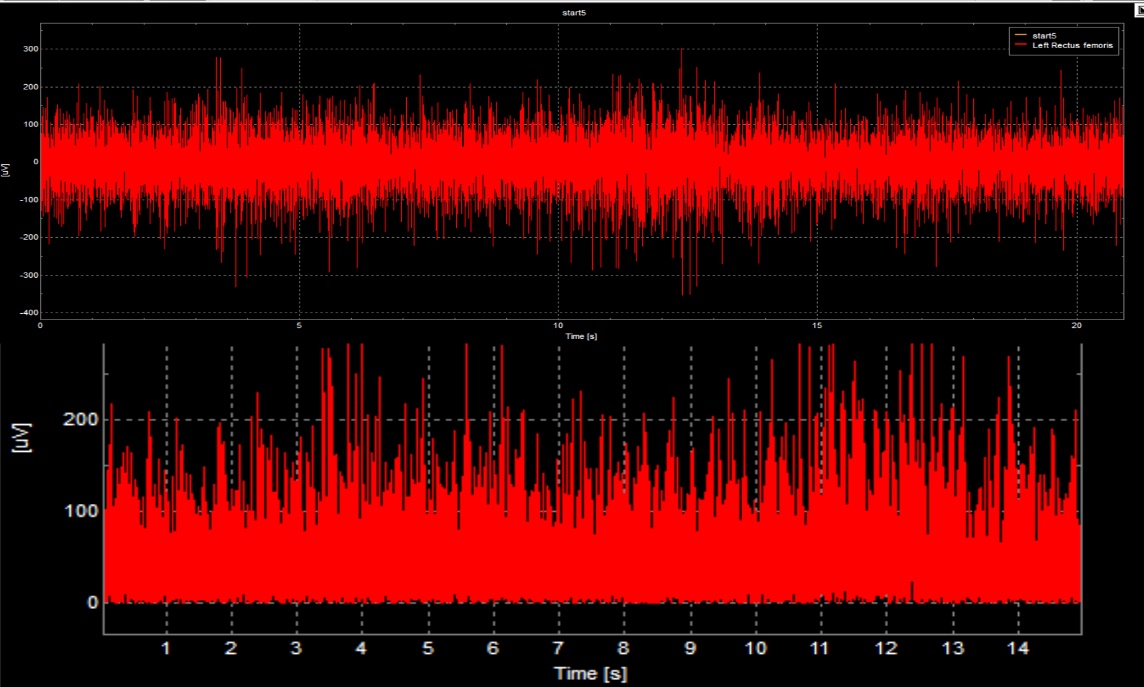


Raw sEMG signal acquisition, interception, filtering, smoothing and other processing

**Table 1.** Correlation between muscle thickness asymmetry index, muscle sEMG asymmetry index during straight leg raising, and VAS score

|  | UG | | BG | |
| --- | --- | --- | --- | --- |
|  | r | p | r | p |
| Asythick(RF) | 0.315 | 0.090 | 0.264 | 0.192 |
| Asythick(VI) | 0.389 | 0.034^*^ | 0.134 | 0.515 |
| Asythick(VM) | 0.453 | 0.012^*^ | 0.384 | 0.053 |
| Asythick(VL) | 0.321 | 0.084 | 0.008 | 0.968 |
| SLR Asy_RMS_(RF) | 0.416 | 0.022^*^ | 0.434 | 0.027^*^ |
| SLR Asy_RMS_(VM) | 0.494 | 0.006^**^ | 0.544 | 0.004^**^ |
| SLR Asy_RMS_(VL) | 0.368 | 0.046^*^ | 0.392 | 0.048^*^ |

*UG: unilateral group; BG: bilateral group;Asythick(RF):asymmetry index of rectus femoris thickness;Asythick(VI):asymmetry index of vastus medialis thickness;Asythick(VM): asymmetry index of vastus medialis thickness;Asythick(VL):asymmetry index of vastus lateralis thickness;SLR:straight leg raising task;Asy-_RMS_(RF):asymmetry index of rectus femoris’s RMS; Asy-_RMS_(VM): asymmetry index of vastus medialis’sRMSs; Asy-_RMS_(VL): asymmetry index of vastus lateralis’sRMS;* indicates P < 0.05; ** indicates P < 0.01.*
